# Using a birth cohort to study brain health and preclinical dementia: recruitment and participation rates in Insight 46

**DOI:** 10.1186/s13104-018-3995-0

**Published:** 2018-12-13

**Authors:** Sarah-Naomi James, Christopher A. Lane, Thomas D. Parker, Kirsty Lu, Jessica D. Collins, Heidi Murray-Smith, Michelle Byford, Andrew Wong, Ashvini Keshavan, Sarah Buchanan, Sarah E. Keuss, Diana Kuh, Nick C. Fox, Jonathan M. Schott, Marcus Richards

**Affiliations:** 1 MRC Unit for Lifelong Health and Ageing at UCL, London, UK; 2 Dementia Research Centre, UCL Institute of Neurology, University College London, London, UK

**Keywords:** Participation, Birth cohort, Longitudinal study, Neuroimaging, Sub-study, Older adults, Attrition

## Abstract

**Objective:**

Identifying and recruiting people with early pre-symptomatic Alzheimer’s disease to neuroimaging research studies is increasingly important. The extent to which results of these studies can be generalised depends on the recruitment and representativeness of the participants involved. We now report the recruitment and participation patterns from a neuroscience sub-study of the MRC National Survey of Health and Development, “Insight 46”. This study aimed to recruit 500 participants for extensive clinical and neuropsychological testing, and neuroimaging. We investigate how sociodemographic factors, health conditions and health-related behaviours predict participation at different levels of recruitment.

**Results:**

We met our target recruitment (n = 502). Higher educational attainment and non-manual socio-economic position (SEP) were consistent predictors of recruitment. Health-related variables were also predictive at every level of recruitment; in particular higher cognition, not smoking and better self-rating health. Sex and APOE-e4 status were not predictors of participation at any level. Whilst recruitment targets were met, individuals with lower SEP, lower cognition, and more health problems are under-represented in Insight 46. Understanding the factors that influence recruitment are important when interpreting results; for Insight 46 it is likely that health-related outcomes and life course risks will under-estimate those seen in the general population.

**Electronic supplementary material:**

The online version of this article (10.1186/s13104-018-3995-0) contains supplementary material, which is available to authorized users.

## Introduction

There is growing interest in recruiting people with early pre-symptomatic Alzheimer’s disease (AD) into neuroimaging studies [1]. Successful recruitment of older adults into dementia studies is notably challenging [2–4]; efforts are being made to create registers and “ready-made” cohorts, which includes embedding sub-studies within existing longitudinal studies [5–7]. To plan relevant studies, and interpret whether results are generalisable, it is important to understand factors that influence recruitment and retention [8, 9]. For example, educational and socioeconomic disadvantage are well established factors of drop out in longitudinal population-based studies [8–10].

The Medical Research Council National Survey of Health and Development (NSHD) is the longest running British birth cohort and has assessed individuals from birth [11]. At the 23rd follow-up at age 60–64, the cohort sample has remained broadly representative of the general population [8]. Over the first 69 years of follow-up, participation rates have varied between 78 and 94% [11, 12], and have not declined with age, but those of lower SEP and cognition are less likely to participate [8, 11]. Here we provide an overview of recruitment and participation in Insight 46, a detailed neuroscience sub-study of NSHD.

## Main text

### Methods

#### Procedure

The NSHD is a representative sample of 5362 males and females who were born in England, Scotland and Wales in 1 week in March 1946. The 24th data collection was conducted at age 68–69 years [11]. Insight 46 has been described in detail elsewhere [7]. In brief, 502 participants attended a clinic in University College London where they took part in a whole day of testing. Participants underwent neuropsychological and neurological examination and 60-min scanning session, with simultaneous collection of dynamic β-amyloid PET (370 MBq florbetapir F18) and MRI. Ethical approval for Insight 46 was granted by the National Research Ethics Service (NRES) Committee London (14/LO/1173).

#### Recruitment

**Fig. 1:**
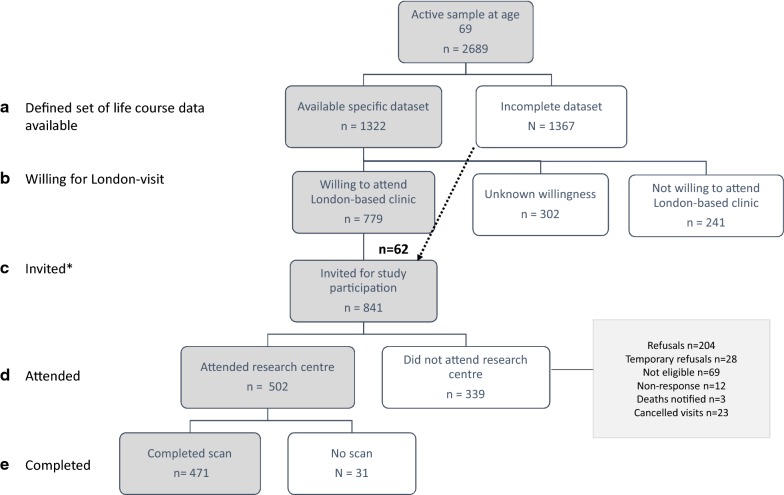
Deriving the sample for the neuroscience sub-study of the MRC National Survey of Health and Development, Insight 46, at age 69–71. *Eligibility for recruitment was considered if participants met the criteria of having a specific set of life course data available (outlined in Additional file 1: Table S3) and previously indicated they may be willing to attend a London-based clinic

The first stage of recruitment consisted of identifying NSHD participants who had not previously withdrawn, died, or remained untraced from the main study by age 69 (Fig. 1, max n = 2698). Participants were asked if they were willing to take part in a smaller clinical trial (yes = 40%), and if so, travel if this clinic was in London (yes = 70%).

Participants were defined as eligible for recruitment to Insight 46 if they met the criteria of having a defined set of life course data available (outlined in Additional file 1: Table S3 [7]) (Fig. 1a); and expressed willingness to come to a London-based clinic visit (Fig. 1b). Invitations were sent to 779 eligible participants (Fig. 1c). To reach our target sample of 500, towards the end of recruitment we relaxed for key life course data to include participants without a previous measure of lung function, smoking or physical exercise (n = 62). Invited participants were screened by telephone, and attended the research centre (n = 502, Fig. 1d). Participants were excluded if they had contraindications to MRI or PET, such as severe claustrophobia, or implantable devices such as pacemakers and intracranial clips [7]. Of the 502 participants, 471 completed scanning (Fig. 1e).

#### Predictors of participation

We investigated socioeconomic and health-related characteristics previously associated with participation [8, 11]. Childhood socioeconomic position (SEP) was derived from paternal occupation; adult SEP was derived from participants’ own occupation at 53 years. SEP was dichotomised into manual (skilled manual, semi-skilled and unskilled) or non-manual (professional, intermediate, skilled non-manual) professions. The highest educational attainment achieved by 26 years was categorised into: no qualification; vocational only, ordinary (‘O’) level or equivalent; and advanced (‘A’) level or equivalent, or higher [13]. Childhood cognitive function was derived from four tests of verbal and non-verbal ability [14]. Adult verbal memory was captured by a word list learning test at age 69 [15]. Cognitive scores were grouped [8, 16] into lowest 10%, middle 80% and highest 10%. APOE-e4 status derived from a blood sample at age 53 [17] was categorised as no ε4; heterozygous ε4; and homozygous ε4. The remaining measures were obtained at ages 68–69. Affective symptoms were measured using the 28-item version of the General Health Questionnaire [18] and a validated threshold indicated severity consistent with a “mental health disorder”. Lifetime smoking years was recoded as: never, ex and current smoker. Alcohol use was recorded as: never, less than once a week, 2–3 times per week, or 4+ times per week. Based on measured height and weight, participants were classified as not overweight (body mass index (BMI) < 25.0 kg/m^2^), overweight (BMI of 25.0 to < 30.0 kg/m^2^), or obese (BMI > 30.0 kg/m^2^). Type II diabetes was based on self-report of doctor diagnosis or use of diabetic medication up to age 69. Hypertension was based on self-report of doctor diagnosis. Overall disease burden was previously derived [11]. Participants self-rated their health as poor, fair, good, very good or excellent.

To derive residence distance from London we calculated the straight-line distance in miles between participants’ post codes recorded in 2016 to the postcode of our London-based Research Centre. Distance was categorised as: < 60, 60–120, 120–180, or > 180 miles.

#### Analysis

We investigated how sociodemographic and health-related characteristics differed in four stages of recruitment and participation (Fig. 1): (1) between those with key life course data vs. those not; of those, (2) between those willing to attend the clinic vs. not; of those (3) between those who attended vs. those who did not; of those (4) between those who completed neuroimaging vs. those who did not. Multivariable logistic regression models estimated associations between predictors and the above stages. Models were initially unadjusted (Table 1), then adjusted for sex, education, childhood and adult SEP (Table 2).

**Table 1:** Analyses of socioeconomic and health characteristics predictors for different levels of recruitment for Insight 46, with no adjustments

Variable	A. Specific life course data available Max n = 2689			B. Willingness for London-clinic Max n = 1322			C. Attended Max n = 841			D. Scanned Max n = 502		
	OR	P	95% CI	OR	P	95% CI	OR	P	95% CI	OR	P	95% CI
Sex												
Female	Reference			Reference			Reference			Reference		
Male	1.19	*0.03*	1.02, 1.38	0.92	0.48	0.74, 1.15	0.77	0.07	0.59, 1.02	0.89	0.76	0.43, 1.85
Childhood SEP												
Manual	Reference			Reference			Reference			Reference		
Non-manual	1.58	< *0.01*	1.35, 1.85	1.57	*< 0.01*	1.26, 1.96	1.18	0.24	0.90, 1.56	2.04	0.06	0.96, 4.33
Educational attainment to age 26												
None	Reference			Reference			Reference			Reference		
Up to GCE (age 16)	2.03	< *0.01*	1.66, 2.48	1.55	< *0.01*	1.15, 2.08	1.74	*0.01*	1.16, 2.61	0.85	0.80	0.25, 2.86
A-level and above (age 16+)	3.09	< *0.01*	2.55, 3.73	2.29	*< 0.01*	1.74, 3.02	1.76	*< 0.01*	1.22, 2.54	0.77	0.64	0.25, 2.33
Adult SEP												
Manual	Reference			Reference			Reference			Reference		
Non-manual	2.22	*< 0.01*	1.88, 2.63	1.88	*< 0.01*	1.45, 2.43	1.70	*< 0.01*	1.20, 2.42	1.08	0.87	0.40, 2.92
Childhood cognitive score												
Bottom 10%	Reference			Reference			Reference			Reference		
Middle 80%	2.84	*< 0.01*	2.08, 3.88	2.60	*< 0.01*	1.52, 4.45	1.45	0.35	0.66, 3.18	1.20	0.87	0.15, 9.56
Top 10%	3.23	*< 0.01*	2.19, 4.75	3.10	*< 0.01*	1.67, 5.75	1.90	0.15	0.80, 4.49	1.97	0.57	0.19, 20.56
Word learning test memory score at 69 years												
Bottom 10%	Reference			Reference			Reference			Reference		
Middle 80%	1.64	*< 0.01*	1.24, 2.17	1.51	*0.05*	1.00, 2.27	1.60	0.10	0.92, 2.80	1.21	0.80	0.27, 5.41
Top 10%	2.45	*< 0.01*	1.63, 3.69	1.78	*0.04*	1.04, 3.03	2.11	*0.04*	1.05, 4.23	1.49	0.67	0.23, 9.50
Mental health prevalence at age 69												
No	Reference			Reference			Reference			Reference		
Yes	0.71	*0.01*	0.56, 0.91	0.77	0.14	0.55, 1.09	0.42	< *0.01*	0.27, 0.66	0.30	*0.01*	0.11, 0.78
Lifetime smoking by 69 years												
Never smoker	Reference			Reference			Reference			Reference		
Ex-smoker	0.94	0.47	0.79, 1.12	0.90	0.38	0.70, 1.14	1.00	1.00	0.74, 1.35	0.48	0.12	0.19, 1.20
Current smoker	0.51	*< 0.01*	0.38, 0.68	0.54	*0.01*	0.34, 0.86	0.43	*0.01*	0.23, 0.82	0.34	0.15	0.05, 1.58
Alcohol use at age 69												
Never	Reference			Reference			Reference			Reference		
Less than once a week	1.17	0.27	0.89, 1.54	0.93	0.72	0.64, 1.37	1.32	0.27	0.81, 2.16	0.32	0.18	0.03, 1.95
2–3× per week	1.38	*0.03*	1.03, 1.85	1.48	0.06	0.99, 2.21	2.16	*< 0.01*	1.30, 3.59	0.36	0.35	0.04, 2.96
4+ times per week	1.37	*0.03*	1.03, 1.83	1.36	0.12	0.92, 2.01	1.51	0.10	0.93, 2.47	0.44	0.44	0.05, 3.58
Weight status at age 69												
Underweight or normal	Reference			Reference			Reference			Reference		
Overweight	0.70	*< 0.01*	0.57, 0.87	0.83	0.17	0.63, 1.08	1.59	*0.01*	1.14, 2.22	0.50	0.25	0.16, 1.61
Obese	0.59	*< 0.01*	0.47, 0.74	0.83	0.23	0.62, 1.12	1.33	0.12	0.93, 1.92	0.22	*0.01*	0.07, 0.69
Type II diabetes by age 69												
No	Reference			Reference			Reference			Reference		
Yes	0.69	*< 0.01*	0.54, 0.88	0.95	0.79	0.65, 1.38	1.18	0.49	0.73, 1.91	0.76	0.62	0.25, 2.27
Hypertension by age 69												
No	Reference			Reference			Reference			Reference		
Yes	0.60	*< 0.01*	0.51, 0.71	0.83	0.11	0.66, 1.04	0.92	0.54	0.69, 1.21	1.05	0.89	0.50, 2.19
Overall disease burden by age 69												
None	Reference			Reference			Reference			Reference		
1	0.87	0.27	0.69, 1.11	1.36	*0.05*	1.00, 1.84	0.82	0.29	0.56, 1.19	1.09	0.86	0.42, 2.85
2	0.81	0.12	0.62, 1.06	1.00	1.00	0.71, 1.41	0.86	0.50	0.55, 1.33	0.98	0.97	0.33, 2.91
3+	0.67	*< 0.01*	0.51, 0.88	1.14	0.47	0.80, 1.63	0.59	*0.02*	0.38, 0.92	0.74	0.59	0.25, 2.21
Self-rated health at age 68												
Poor	Reference			Reference			Reference			Reference		
Fair	1.22	0.45	0.73, 2.02	1.43	0.38	0.64, 3.19	1.49	0.54	0.42, 5.22	0.35	0.19	0.07, 1.67
Good	1.57	0.07	0.97, 2.54	1.64	0.20	0.77, 3.51	2.97	0.08	0.89, 9.92	0.61	0.46	0.16, 2.29
Very good	2.49	*< 0.01*	1.54, 4.04	2.11	*0.05*	0.99, 4.47	4.39	*0.02*	1.33, 14.54	0.80	0.74	0.22, 2.88
Excellent	2.34	*< 0.01*	1.37, 4.00	2.98	*0.01*	1.31, 6.79	4.18	*0.03*	1.20, 14.55	1.00	-	1.00, 1.00
APOE status												
No e4	Reference			Reference			Reference			Reference		
e4 heterozygous	0.87	0.19	0.72, 1.07	0.99	0.96	0.75, 1.31	0.98	0.93	0.69, 1.41	1.16	0.76	0.45, 2.95
e4 homozygous	1.26	0.37	0.76, 2.07	0.70	0.28	0.37, 1.33	0.91	0.83	0.36, 2.25	0.76	0.80	0.09, 6.21
Residential distance from London centre (miles)												
< 60	Reference			Reference			Reference			Reference		
60–120	0.92	0.42	0.75, 1.13	0.68	*0.02*	0.50, 0.93	1.35	0.09	0.95, 1.93	NA		
120–180	0.91	0.39	0.74, 1.13	0.39	*< 0.01*	0.29, 0.54	1.13	0.53	0.77, 1.67			
> 180	0.86	0.17	0.69, 1.07	0.38	*< 0.01*	0.27, 0.52	1.07	0.75	0.71, 1.60			

SEP: socioeconomic position; OR: odds ratio; A: Comparing those who did not meet the specific life course data availability criteria vs those who did; B: comparing those who previously indicated they were not willing to attend a London-based clinic or did not respond vs those who were; C: comparing those who did not attend vs successfully attended; D: comparing those who were not scanned vs those successfully scanned

Italic values indicate p < 0.05

**Table 2:** Socioeconomic and health characteristics predictors of levels of recruitment for Insight 46, adjusting for sex, childhood and adult SEP and education

Variable	A. Specific life course data available			B. Willingness for London-clinic			C. Attended			D. Scanned		
	OR	P	95% CI	OR	P	95% CI	OR	P	95% CI	OR	P	95% CI
Childhood cognitive score												
Bottom 10%	Reference			Reference			Reference			Reference		
Middle 80%	1.55	*0.01*	1.10, 2.19	1.66	0.07	0.96, 2.90	1.06	0.89	0.45, 2.48	1.26	0.84	0.14, 11.54
Top 10%	1.06	0.79	0.68, 1.65	1.78	0.09	0.92, 3.44	1.28	0.60	0.50, 3.30	2.07	0.57	0.17, 25.65
Word learning test memory score at 69 years												
Bottom 10%	Reference			Reference			Reference			Reference		
Middle 80%	1.25	0.16	0.92, 1.70	1.27	0.28	0.82, 1.94	1.40	0.26	0.79, 2.49	1.15	0.86	0.25, 5.29
Top 10%	1.56	0.07	0.97, 2.50	1.25	0.45	0.71, 2.21	1.77	0.13	0.85, 3.68	1.32	0.78	0.19, 8.94
Mental health prevalence at age 69												
No	Reference			Reference			Reference			Reference		
Yes	0.78	0.07	0.59, 1.02	0.91	0.60	0.63, 1.30	0.46	*< 0.01*	0.29, 0.73	0.28	*0.01*	0.10, 0.75
Lifetime smoking to 69 years												
Never smoker	Reference			Reference			Reference			Reference		
Ex-smoker	0.98	0.86	0.81, 1.19	0.90	0.42	0.70, 1.16	0.99	0.97	0.73, 1.35	0.47	0.11	0.18, 1.18
Current smoker	0.64	*0.01*	0.46, 0.88	0.70	0.14	0.43, 1.12	0.48	*0.03*	0.25, 0.94	0.33	0.21	0.06, 1.86
Alcohol use at age 69												
Never	Reference			Reference			Reference			Reference		
Less than once a week	1.09	0.58	0.80, 1.48	0.91	0.63	0.61, 1.35	1.28	0.34	0.77, 2.13	0.32	0.29	0.04, 2.62
2–3× per week	1.21	0.25	0.87, 1.68	1.22	0.35	0.80, 1.85	1.91	*0.02*	1.13, 3.25	0.45	0.46	0.05, 3.73
4+ times per week	1.17	0.35	0.84, 1.61	1.11	0.62	0.74, 1.67	1.29	0.33	0.77, 2.16	0.54	0.57	0.06, 4.63
Overweight at age 69												
No	Reference			Reference			Reference			Reference		
Overweight	0.74	*0.01*	0.58, 0.94	0.89	0.40	0.67, 1.17	1.61	*0.01*	1.14, 2.28	0.39	0.15	0.10, 1.43
Obese	0.67	*< 0.01*	0.52, 0.86	0.95	0.72	0.70, 1.28	1.45	*0.05*	0.99, 2.12	0.17	*0.01*	0.05, 0.62
Type II diabetes at age 69												
No	Reference			Reference			Reference			Reference		
Yes	0.83	0.18	0.63, 1.09	1.04	0.85	0.70, 1.53	1.21	0.46	0.73, 2.01	0.68	0.50	0.22, 2.06
Hypertension by age 69												
No	Reference			Reference			Reference			Reference		
Yes	0.61	*< 0.01*	0.51, 0.73	0.86	0.21	0.69, 1.09	0.92	0.58	0.69, 1.23	0.98	0.94	0.46, 2.07
Overall disease burden at age 69												
None	Reference			Reference			Reference			Reference		
1	0.81	0.13	0.62, 1.06	1.31	0.09	0.96, 1.78	0.79	0.24	0.54, 1.16	1.18	0.74	0.45, 3.12
2	0.70	*0.02*	0.52, 0.94	1.01	0.94	0.71, 1.44	0.84	0.44	0.53, 1.31	1.15	0.81	0.36, 3.67
3+	0.72	*0.03*	0.53, 0.97	1.23	0.28	0.85, 1.77	0.63	*0.04*	0.40, 0.99	0.71	0.54	0.23, 2.15
Self-rated health at age 68												
Poor	Reference			Reference			Reference			Reference		
Fair	1.05	0.85	0.62, 1.80	1.35	0.47	0.59, 3.08	1.40	0.61	0.39, 5.05	0.37	0.22	0.08, 1.81
Good	1.30	0.31	0.78, 2.17	1.36	0.44	0.62, 2.97	2.66	0.12	0.77, 9.15	0.61	0.47	0.16, 2.35
Very good	1.93	*0.01*	1.16, 3.22	1.65	0.21	0.76, 3.58	3.75	*0.04*	1.10, 12.77	0.91	0.88	0.25, 3.32
Excellent	1.97	*0.02*	1.11, 3.50	2.07	0.09	0.89, 4.82	3.42	0.06	0.95, 12.29	1.00	-	1.00, 1.00
APOE status												
No e4	Reference			Reference			Reference			Reference		
e4 heterozygous	0.85	0.13	0.68, 1.05	0.99	0.97	0.75, 1.32	0.98	0.93	0.68, 1.42	1.08	0.88	0.41, 2.80
e4 homozygous	1.27	0.39	0.73, 2.21	0.79	0.50	0.40, 1.56	0.97	0.95	0.38, 2.46	0.66	0.70	0.08, 5.46
Residential distance to London centre (miles)												
< 60	Reference			Reference			Reference			Reference		
60–120	0.99	0.92	0.79, 1.24	0.65	*0.01*	0.48, 0.90	1.21	0.09	0.96, 2.16	NA		
120–180	0.99	0.91	0.78, 1.24	0.40	*< 0.01*	0.29, 0.55	1.19	0.39	0.80, 1.78			
> 180	0.87	0.24	0.68, 1.10	0.36	*< 0.01*	0.26, 0.50	1.11	0.62	0.73, 1.68			

SEP: socioeconomic position; OR: odds ratio. A: Comparing those that didn’t meet the specific life course data availability criteria vs those who did; B: comparing those that previously indicated they were not willing to attend a London-based clinic or didn’t respond vs those who were; C: comparing those that didn’t attend vs successfully attended; D: comparing those who were not scanned vs those successfully scanned

Italic values indicate p < 0.05

### Results

Distributions of predictors at each level are shown in Additional file 1: Table S1.

#### Those with key life course data

Of 2689 participants initially identified, 1322 (50%) had key life course data and previously attended a clinic (Fig. 1a). Eligibility was associated with non-manual childhood and adult SEP and higher education (Table 1) not with sex. Those eligible had higher cognitive performance, alcohol intake and self-rated health; and lower lifetime smoking, and fewer mental and other health problems. Adjustment for sex, education and SEP slightly attenuated some of these results (Table 2). Although the bigger predictor was being in the highest 10% of childhood cognitive scores (OR = 3.23 (95% CI 2.19, 4.75)), this was attenuated substantially by adjustment for other early factors (OR = 1.06 (0.68, 1.65) Table 2). There was no difference in eligibility by APOE-e4 status.

#### Those willing to attend a London-based assessment

Of the above 1322 eligible, 779 (59%) indicated willingness to attend a London clinic, 302 (23%) did not indicate a preference, and 241 (18%) declined (Fig. 1b). As with eligibility, non-manual SEP and higher education, but not gender, were associated with higher likelihood of this willingness. Higher cognitive performance and non-smoking were predictors of willingness, although these differences were largely attenuated after adjustment for early factors (Table 2). While there was limited evidence for differences in mental health, health and health-related behaviours and APOE-e4 status between those willing and not willing to attend, those who rated their health as “excellent” were more likely to be willing (OR = 2.98 (1.31, 6.79)). Having a residential address the furthest away from the research centre was associated with lower willingness (OR = 0.38 (0.27, 0.52)).

#### Those invited into the study

All the above 779 were invited (Fig. 1c). When recruitment was underway the life course data criterion was relaxed to include 62 participants without a previous measure of lung function, smoking or physical exercise.

#### Those who attended the research centre

Of the 841 invited, 502 (60%) attended the clinic; 204 (24%) refused, 28 (3%) temporarily refused, 12 (1%) did not respond, 3 (0.4%) died, 23 (3%) cancelled visits and 69 (8%) were excluded (Fig. 1d) for reasons including severe claustrophobia (n = 34) and metal implants (n = 28). Mean age at testing was 70.7 years (range 69.2 to 71.9), as expected for this age-homogenous group. Residential distance from the clinic ranged from 2 to 467 miles (mean = 112.6). Those who attended rated high visit satisfaction (over 98%).

Higher educational attainment and non-manual SEP were predictors of attendance. The strongest predictor was a health self-rating of “very good” (OR = 4.39 (1.33, 14.54)). Notably, greater distance from the clinic did not predict attendance (OR = 1.07 (0.71, 1.60)). We limited this analysis by comparing those who attended with those who refused; the pattern remained similar (Additional file 1: Table S2).

#### Those who had complete scans

Of the 502 who attended, 471 (94%) completed the PET/MRI scan. Reasons for not completing were claustrophobia (n = 25); PET/MRI incompatibility issues (n = 4); recent illness (n = 1); withdrawal before being rescheduled (n = 1; from n = 51 rescheduled scans). The direction of predictors of completion were mostly similar to previously observed; significant predictors were not having concurrent mental health problems (OR = 0.30) (0.11, 0.78) and not being obese (OR = 0.22) (0.07, 0.69) (Table 2).

### Discussion

We provide a detailed overview of recruitment for an observational specialised (neuroscience) sub-study, embedded in the longest running British birth cohort, now in its early 7th decade. Participation requires a full day visit of intensive phenotyping and neuroimaging, and in many cases considerable travel (mean travel distance = 112.55 miles) and overnight stay away from home (78%) [19]. Despite this, recruitment targets were met (original target n = 500, achieved target n = 502) and 98% of those attended with high satisfaction.

However, even in a sample already biased towards higher SEP and education [8, 11], higher education and non-manual SEP in adulthood were independent and consistent predictors of recruitment at every stage; for example, from those who were invited, those from a non-manual adult SEP had 57% higher odds of attending the study compared to those from manual SEP (Table 2). Health-related characteristics, particularly higher cognition, were also predictive of every stage of recruitment, although these effects were largely attenuated when adjusting for education and SEP. Reasons why lower childhood cognition, SEP and education are constant predictors of attrition may include reduced understanding or consideration of the importance of research, decreased confidence in participation, or concerns about performance [8, 10].

Those who attended were less likely to be a current smoker, or to be obese; and had fewer clinical disorders, better mental health, and “very good” or “excellent” self-rated health. This may reflect those with healthier lifestyles and better health being more likely to be interested in health-related research, or may be more able to cope with the demands of travel and the assessments.

Notably however, sex and APOE-e4 genotype, an important predictor of β-amyloid load [20] and AD [21], were not predictors of participation at any level. The sex and APOE-e4 ratio are similar to national rates in England and Wales [22, 23].

The procedure and set-up of Insight 46 has similar demands to those of some preclinical AD trials; thus our results should prove useful in the design, interpretation and generalisability of similar studies seeking to embed preclinical AD studies or trials. The rich sociodemographic and health-related behaviour measures collected prospectively over the life course in NSHD further allowed for a systematic assessment of predictors of participation.

In summary, we show that recruitment to and participation in a neuroscience sub-study of a population-based cohort is associated with bias towards higher SEP, education, cognitive function, and better health. Those at highest risk for negative outcomes may be under-represented in Insight 46. In many cases longitudinal studies such as this offer opportunities for assessing relationships between exposures and health outcomes across the life course, which may not require participants to be representative of the population at large [24, 25]. For studies that aim to be representative, associations with health-related outcomes may underestimate the strength of associations in the wider population, which needs to be considered when interpreting results. Nevertheless, it is equally of interest to investigate pre-clinical findings in a sample of lower risk.

## Limitations

Our findings are based on a generation of British participants in their early 7th decade, who are part of a lifelong study which may not directly generalise to younger populations, or populations outside of existing studies, where there may be less motivation to participate. In addition, our predictors of participation may be specific to single centre neuroimaging studies given that some people were only excluded due to unwillingness to travel sometimes long distances. The data we describe have only related to cross-sectional recruitment. We are currently undertaking longitudinal follow-up of individuals which will enable investigation of predictors of study retention.

## Additional file

**Additional file 1: Table S1.** Numbers and percentage of socioeconomic factors and health characteristics distribution for different stages of recruitment. **Table S2.** Analyses of socioeconomic and health characteristics predictors for those who were invited but refused attendance vs those who attended. **Table S3.** Original criteria of set of life course data available for Insight 46 eligibility.

## Abbreviations

AD: Alzheimer's disease
MRC: Medical Research Council
NSHD: National Survey of Health and Development
PET: positron emission tomography
MRI: magnetic resonance imaging
SEP: socioeconomic position
OR: odds ratio
