# Supplementary material for: Using a birth cohort to study brain health and preclinical dementia: recruitment and participation rates in Insight 46
Source: BMC Res Notes. 2018 Dec 13;11:885. doi: 10.1186/s13104-018-3995-0 (PMC6293512; doi:10.1186/s13104-018-3995-0)
Supplement: Supplementary file 1 — Additional file 1: Table S1. Numbers and percentage of socioeconomic factors and health characteristics distribution for different stages of recruitment. Table S2. Analyses of socioeconomic and health characteristics predictors for those who were invited but refused attendance vs those who attended. Table S3. Original criteria of set of life course data available for Insight 46 eligibility. [file 13104_2018_3995_MOESM1_ESM.docx]

Additional Files for “Using a birth cohort to study brain health and preclinical dementia: Recruitment and participation rates in Insight 46”

**Table S1: Numbers and percentage of socioeconomic factors and health characteristics distribution for different stages of recruitment.**

| Variable |  | A. Dataset available | | | B. Willingness to London-clinic | | C. Attended | |
| --- | --- | --- | --- | --- | --- | --- | --- | --- |
|  |  | Yes | No | Yes | | No/unknown | Yes | No |
|  | Max n | 1322 | 1367 | 779 | | 543 | 502 | 339 |
| Sex |  |  |  |  | |  |  |  |
|  | Female | 694 (51%) | 615 (46%) | 369 (48%) | | 247 (45%) | 256 (51%) | 151 (44%) |
| Childhood SEP | |  |  |  | |  |  |  |
|  | Manual | 650 (50%) | 758 (61%) | 424 (55%) | | 233 (44%) | 216 (44%) | 160 (47%) |
| Educational attainment to age 26 | |  |  |  | |  |  |  |
|  | None | 323 (24%) | 559 (45%) | 150 (19%) | | 173 (32%) | 78 (16%) | 81 (25%) |
|  | Up to GCE | 391 (39%) | 334 (27%) | 224 (29%) | | 167 (31%) | 151 (31%) | 90 (27%) |
|  | A-level and above | 608 (46%) | 341 (28%) | 405 (52%) | | 204 (38%) | 273 (55%) | 161 (49%) |
| Adult SEP | |  |  |  | |  |  |  |
|  | Manual | 306 (23%) | 534 (40%) | 635 (81%) | | 381 (70%) | 76 (15%) | 79 (23%) |
| Childhood cognitive score | |  |  |  | |  |  |  |
|  | Bottom 10% | 61 (5%) | 142 (12%) | 22 (3%) | | 39 (7%) | 13 (3%) | 13 (4%) |
|  | Middle 80% | 1107 (83%) | 908 (78%) | 659 (85%) | | 449 (83%) | 415 (83%) | 286 (85%) |
|  | Top 10% | 154 (12%) | 111 (10%) | 98 (13%) | | 56 (10%) | 74 (15%) | 39 (12%) |
| Adult | |  |  |  | |  |  |  |
|  | Bottom 10% | 103 (8%) | 118 (14%) | 53 (7%) | | 50 (11%) | 27 (6%) | 27 (9%) |
|  | Middle 80% | 983 (81%) | 688 (80%) | 605 (82%) | | 378 (80%) | 404 (82%) | 252 (82%) |
|  | Top 10% | 124 (10%) | 58 (7%) | 81 (11%) | | 43 (9%) | 59 (12%) | 28 (9%) |
| Mental health prevalence at age 69 | |  |  |  | |  |  |  |
|  | Yes | 153 (12%) | 150 (17%) | 85 (11%) | | 68 (14%) | 38 (8%) | 52 (16%) |
| Lifetime smoking to 69 years | |  |  |  | |  |  |  |
|  | Never smoker | 412 (32%) | 343 (29%) | 257 (34%) | | 156 (30%) | 169 (34%) | 109 (33%) |
|  | Ex smoker | 785 (61%) | 697 (59%) | 468 (61%) | | 317 (61%) | 307 (62%) | 198 (59%) |
|  | Current smoker | 91 (7%) | 149 (13%) | 43 (6%) | | 48 (9%) | 18 (4%) | 27 (8%) |
| Alcohol use at age 69 | |  |  |  | |  |  |  |
|  | Never | 146 (12%) | 133 (15%) | 83 (11%) | | 63 (13%) | 44 (9%) | 43 (14%) |
|  | Less than once a week | 399 (33%) | 311 (35%) | 220 (29%) | | 179 (37%) | 139 (28%) | 103 (33%) |
|  | 2-3 x per week | 312 (25%) | 206 (23%) | 206 (28%) | | 106 (22%) | 150 (31%) | 68 (22%) |
|  | 4+ per week | 374 (31%) | 248 (28%) | 240 (32%) | | 134 (28%) | 158 (32%) | 102 (32%) |
| Overweight at age 69 | |  |  |  | |  |  |  |
|  | No | 400 (32%) | 213 (24%) | 256 (34%) | | 144 (30%) | 148 (30%) | 122 (39%) |
|  | Overweight | 491 (40%) | 373 (42%) | 392 (39%) | | 199 (41%) | 210 (45%) | 109 (35%) |
|  | Obese | 340 (28%) | 305 (35%) | 203 (27%) | | 137 (29%) | 136 (28%) | 84 (27%) |
| Type II Diabetes at age 69 | |  |  |  | |  |  |  |
|  | Yes | 126 (10%) | 155 (14%) | 75 (10%) | | 51 (10%) | 50 (9%) | 29 (10%) |
| Hypertension by age 69 | |  |  |  | |  |  |  |
|  | Yes | 569 (44%) | 620 (57%) | 328 (43%) | | 242 (47%) | 214(43%) | 147 (45%) |
| Overall disease burden at age 69 | |  |  |  | |  |  |  |
|  | None | 317 (27%) | 182 (23%) | 187 (26%) | | 130 (29%) | 131 (27%) | 68 (23%) |
|  | 1 | 408 (35%) | 268 (34%) | 270 (37%) | | 138 (31%) | 178 (37%) | 113 (37%) |
|  | 2 | 234 (20%) | 166 (20%) | 138 (10%) | | 96 (22%) | 96 (20%) | 58 (19%) |
|  | 3+ | 214 (18%) | 183 (23%) | 133 (18%) | | 81 (18%) | 74 (16%) | 65 (21%) |
| Self-rated health at age 69 | |  |  |  | |  |  |  |
|  | Poor | 29 (2%) | 47 (5%) | 13 (2%) | | 16 (4%) | 4 (1%) | 9 (3%) |
|  | Fair | 145 (12%) | 194 (18%) | 78 (10%) | | 67 (14%) | 33 (7%) | 50 (16%) |
|  | Good | 382 (31%) | 395 (37%) | 219 (29%) | | 164 (34%) | 136 (28%) | 103 (32%) |
|  | Very good | 548 (44%) | 356 (32%) | 346 (46%) | | 202 (41%) | 246 (51%) | 126 (39%) |
|  | Excellent | 130 (11%) | 90 (8%) | 92 (12%) | | 38 (8%) | 65 (14%) | 35 (11%) |
| APOE status | |  |  |  | |  |  |  |
|  | No e4 | 825 (73%) | 700 (72%) | 484 (73%) | | 341 (72%) | 323 (73%) | 195 (73%) |
|  | e4 Heterozygous | 264 (24%) | 256 (26%) | 155 (24%) | | 110 (23%) | 106 (24%) | 65 (24%) |
|  | e4 Homozygous | 40 (3%) | 27 (3%) | 20 (3%) | | 20 (4%) | 12 (3%) | 8 (3%) |
| Residential Distance to London Centre | |  |  |  | |  |  |  |
|  | <60 miles | 393 (30%) | 376 (28%) | 280 (36%) | | 114 (21%) | 170 (36%) | 129 (38%) |
|  | 60-120 miles | 340 (26%) | 354 (26%) | 213 (28%) | | 127 (24%) | 148 (30%) | 83 (25%) |
|  | 120-180 miles | 302 (23%) | 317 (24%) | 148 (19%) | | 154 (29%) | 97 (19%) | 65 (19%) |
|  | >180 miles | 273 (21%) | 304 (22%) | 131 (17%) | | 142 (26%) | 83 (17%) | 59 (18%) |

SEP**=**socioeconomic position. A = Comparing those that didn’t meet the specific life course data availability criteria vs those who did; B = Comparing those that previously indicated they were not willing to attend a London-based clinic or didn’t respond vs those who were; C = comparing those that didn’t attend vs successfully attended.

**Table S2: Analyses of socioeconomic and health characteristics predictors for those who were invited but refused attendance vs those who attended.**

| Variable |  | Refusal vs attend | | |
| --- | --- | --- | --- | --- |
|  |  | OR | P | 95% CI |
| Sex | |  |  |  |
|  | Female | Reference | | |
|  | Male | 0.70 | **0.03** | 0.50-0.97 |
| Childhood social class | |  |  |  |
|  | Manual | Reference | |  |
|  | Non-manual | 1.13 | 0.46 | 0.82-1.57 |
| Educational attainment to age 26 | |  |  |  |
|  | None | Reference | |  |
|  | Up to GCE (age 16) | 1.48 | 0.10 | 0.93-2.38 |
|  | A-level and above (age 16+) | 1.69 | **0.02** | 1.10-2.61 |
| Social class at age 53 | |  |  |  |
|  | Manual | Reference | |  |
|  | Non-manual | 1.87 | **<0.01** | 1.25-2.79 |
| Childhood cognitive score | |  |  |  |
|  | Bottom 10% | Reference | |  |
|  | Middle 80% | 0.91 | 0.86 | 0.32-2.60 |
|  | Top 10% | 1.19 | 0.77 | 0.38-3.67 |
| Episodic memory score at 69 years | |  |  |  |
|  | Bottom 10% | Reference | |  |
|  | Middle 80% | 1.74 | 0.09 | 0.92-3.29 |
|  | Top 10% | 2.19 | 0.06 | 0.97-4.92 |
| Mental health prevalence at age 69 | |  |  |  |
|  | No % | Reference | |  |
|  | Yes % | 0.45 | **<0.01** | 0.27-0.76 |
| Lifetime smoking to 69 years | |  |  |  |
|  | Never smoker | Reference | |  |
|  | Ex-smoker | 1.02 | 0.90 | 0.72-1.46 |
|  | Current smoker | 0.42 | 0.02 | 0.20-0.86 |
| Exercise in last month at age 69 | |  |  |  |
|  | No | Reference | |  |
|  | Yes | 1.01 | 0.98 | 0.35-2.91 |
| Alcohol use at age 69 | |  |  |  |
|  | Never | Reference | |  |
|  | monthly or less | 1.35 | 0.31 | 0.75-2.43 |
|  | 2-3 x per week | 1.78 | 0.06 | 0.98-3.23 |
|  | 4+ times per week | 1.44 | 0.22 | 0.80-2.56 |
| Overweight at age 69 | |  |  |  |
|  | No | Reference | |  |
|  | Overweight | 1.64 | **0.01** | 1.11-2.43 |
|  | Obese | 1.50 | 0.07 | 0.97-2.31 |
| Type II Diabetes at age 69 | |  |  |  |
|  | No | Reference | |  |
|  | Yes | 1.15 | 0.62 | 0.65-2.03 |
| Hypertension by age 69 | |  |  |  |
|  | No | Reference | | |
|  | Yes | 0.85 | 0.52 | 0.68-1.06 |
| Cardiovascular event at age 69 | |  |  |  |
|  | No | Reference | |  |
|  | Yes | 0.84 | 0.53 | 0.50-1.44 |
| Overall disease burden at age 69 | |  |  |  |
|  | None | Reference | |  |
|  | 1 | 0.86 | 0.50 | 0.55-1.34 |
|  | 2 | 0.85 | 0.54 | 0.51-1.42 |
|  | 3+ | 0.71 | 0.22 | 0.42-1.22 |
| Self-rated health at age 68 | |  |  |  |
|  | Poor | Reference | |  |
|  | Fair | 0.85 | 0.84 | 0.18-4.13 |
|  | Good | 1.73 | 0.48 | 0.38-7.97 |
|  | Very good | 2.20 | 0.31 | 0.48-10.02 |
|  | Excellent | 2.22 | 0.32 | 0.46-10.68 |
| APOE status | |  |  |  |
|  | No e4 | Reference | |  |
|  | e4 Heterozygous | 1.01 | 0.96 | 0.66-1.56 |
|  | e4 Homozygous | 1.06 | 0.92 | 0.33-3.35 |
| Residential distance to London centre | |  |  |  |
|  | <60 miles | Reference | |  |
|  | 60-120 miles | 1.25 | 0.29 | 0.83,1.89 |
|  | 120-180 miles | 1.2 | 0.46 | 0.75,1.92 |
|  | >180 miles | 1.03 | 0.90 | 0.64,1.66 |

SEP**=**socioeconomic position; OR=odds ratio.

**Table S3: Original criteria of set of life course data available for Insight 46 eligibility *.**

| 1. Attendance at a clinic visit at age 60–64. |
| --- |
| 1. Parental socioeconomic position: at least one indicator of occupational social class or education. |
| 1. Cognition: memory and processing speed from the 60–64 year collection AND at least one set of measures at either ages 8, 11 or 15. |
| 1. Early physical growth trajectories: birth weight and at least one measure of height and weight at ages 4–15. |
| 1. Educational attainment: highest qualification by age 26. |
| 1. Mental health: teacher ratings of behaviour and temperament at ages 13 or 15, and at least one measure of affective symptoms at ages 36, 43, 53 or 60–64. |
| 1. Blood pressure, lung function, adult height and weight: at least one measure of each at ages 36, 43, 53 or 60–64. |
| 1. Health behaviours: at least one measure of smoking and physical exercise at ages 36, 43, 53 or 60–64. |
| 1. Blood: either age 53 or 60–64 samples. |

* Replicated from our protocol paper: (7). When recruitment was underway the criteria were relaxed to remove the requirement for a measure of lung function, smoking or physical exercise (n=62).
